# Lower extremity outcome measures: considerations for clinical trials in spinal cord injury

**DOI:** 10.1038/s41393-018-0097-8

**Published:** 2018-04-27

**Authors:** Marc Bolliger, Andrew R. Blight, Edelle C. Field-Fote, Kristin Musselman, Serge Rossignol, Dorothy Barthélemy, Laurent Bouyer, Milos R. Popovic, Jan M. Schwab, Michael L Boninger, Keith E. Tansey, Giorgio Scivoletto, Naomi Kleitman, Linda A. T. Jones, Dany H. Gagnon, Sylvie Nadeau, Dirk Haupt, Lea Awai, Chris S Easthope, Björn Zörner, Ruediger Rupp, Dan Lammertse, Armin Curt, John Steeves

**Affiliations:** 1 Spinal Cord Injury Center, University Hospital Balgrist, University Zurich, Zurich, Switzerland; 2 Swiss Center for Clinical Movement Analysis (SCMA), Zurich, Switzerland; 3 Acorda Therapeutics, Ardsley, NY USA; 4 Shepherd Center, Georgia Institute of Technology, School of Biological Sciences, Emory University School of Medicine, Division of Physical Therapy, Atlanta, GA USA; 5 Toronto Rehabilitation Institute, University Health Network, Toronto, ON Canada; 6 Department of Physical Therapy, University of Toronto, Toronto, ON Canada; 7 Department of Neuroscience, Faculty of Medicine, Université de Montréal, Montreal, QC Canada; 8 School of Rehabilitation, Faculty of Medicine, Université de Montréal, and Centre de recherche interdisciplinaire en réadaptation (CRIR), Institut universitaire sur la réadaptation en déficience physique de Montréal (IURDPM) du CIUSSS du Centre-Sud-de-l’Ile-de-Montréal, Montreal, QC Canada; 9 Department of Rehabilitation, Faculty of Medicine, Université Laval, Québec, Canada; 10 Rehabilitation Engineering Laboratory, Toronto Rehabilitation Institute, University Health Network, Toronto, ON Canada; 11 Institute of Biomaterials and Biomedical Engineering, University of Toronto, Toronto, ON Canada; 12 Department of Neurology, Spinal Cord Injury Division and Departments of Neuroscience and Physical Medicine and Rehabilitation, The Neurological Institute, The Ohio State University, Wexner Medical Center, Columbus, OH USA; 13 Department of Physical Medicine and Rehabilitation, University of Pittsburgh & Department of Veterans Affairs, VA Pittsburgh Healthcare System, Pittsburgh, PA USA; 14 Methodist Rehabilitation Center, University of Mississippi Medical Center and Jackson VA Medical Center, Jackson, MS USA; 15 Spinal Cord Unit and Spinal Rehabilitation (SpiRe) laboratory, IRCCS Fondazione S. Lucia, Rome, Italy; 16 Craig H. Neilsen Foundation, Encino, CA USA; 17 School of Rehabilitation, Université de Montréal and Pathokinesiology Laboratory, Centre for Interdisciplinary Research in Rehabilitation, Institut universitaire sur la réadaptation en déficience physique de Montréal, CIUSSS Centre-Sud-de-l’Île-de-Montréal, Montreal, QC Canada; 18 University of British Columbia, Vancouver, BC Canada; 19 Spinal Cord Injury Center, Heidelberg University Hospital, Heidelberg, Germany; 20 Craig Hospital, Englewood, Colorado, University of Colorado School of Medicine, Colorado, USA

## Abstract

**Study Design:**

This is a focused review article.

**Objectives:**

To identify important concepts in lower extremity (LE) assessment with a focus on locomotor outcomes and provide guidance on how existing outcome measurement tools may be best used to assess experimental therapies in spinal cord injury (SCI). The emphasis lies on LE outcomes in individuals with complete and incomplete SCI in Phase II-III trials.

**Methods:**

This review includes a summary of topics discussed during a workshop focusing on LE function in SCI, conceptual discussion of corresponding outcome measures and additional focused literature review.

**Results:**

There are a number of sensitive, accurate, and responsive outcome tools measuring both quantitative and qualitative aspects of LE function. However, in trials with individuals with very acute injuries, a baseline assessment of the primary (or secondary) LE outcome measure is often not feasible.

**Conclusion:**

There is no single outcome measure to assess all individuals with SCI that can be used to monitor changes in LE function regardless of severity and level of injury. Surrogate markers have to be used to assess LE function in individuals with severe SCI. However, it is generally agreed that a direct measurement of the performance for an appropriate functional activity supersedes any surrogate marker. LE assessments have to be refined so they can be used across all time points after SCI, regardless of the level or severity of spinal injury.

**Sponsors:**

Craig H. Neilsen Foundation, Spinal Cord Outcomes Partnership Endeavor.

## Introduction

The assessment of lower extremity (LE) function with a focus on locomotor outcomes may be perceived as straightforward even though LE function encompasses standing, postural control to overground locomotion. Although bipedal human gait may to some extent vary within and between individuals, it adheres to some fundamental characteristics of LE function. Human gait is usually rhythmic, coordinated, alternating, symmetrical, and adaptable to environmental demands. Furthermore, unidimensional measures such as speed and endurance intervals are highly objective ambulatory measures, but have limitations when assessing LE locomotor characteristics.

In pre-clinical animal models of spinal cord injury (SCI) in rodents and cats, the number of experimental therapeutics and/or active rehabilitation training paradigms improving locomotor outcomes is consistently increasing, facilitated by standardized measures for locomotor function and kinematics [1–5]. Some of these promising interventions are currently being investigated in human studies (http://scope-sci.org/trials/). LE outcome measures have been designed to identify the recovery pattern after SCI and to assess specific aspects of LE activity. Each outcome measure has distinct strengths and limitations. In this review, an international panel discussed and debated the merits of the available LE assessments, including their ability to detect clinically meaningful changes over time (responsiveness). The authors provide recommendations for the applicability of these LE outcome measures to clinical trials.

This review was stimulated by a workshop in 2015 that was co-sponsored by the Craig H. Neilsen Foundation (www.chnfoundation.org) and the Spinal Cord Outcomes Partnership Endeavor (www.scope-sci.org). The goal was to identify important concepts in outcome assessment and provide the informed opinions and guidance of the workshop participants on how existing outcome measurement tools may be best used to assess the effectiveness of experimental therapies in SCI. The focus was on LE outcomes in individuals with complete and incomplete SCI in Phase II–III trials where the therapeutic intervention is directed to alter central nervous system function at any level, either below, across, or above the level of injury. Phase II studies assess safety and efficacy (proof of concept) mainly on the level of neurological impairments, while phase III trials focus on effectiveness, efficacy and safety more on a functional thus clinical meaningful level. The different foci of the trial phases result in different outcome measures selection while designing a trial.

**Fig. 1:**
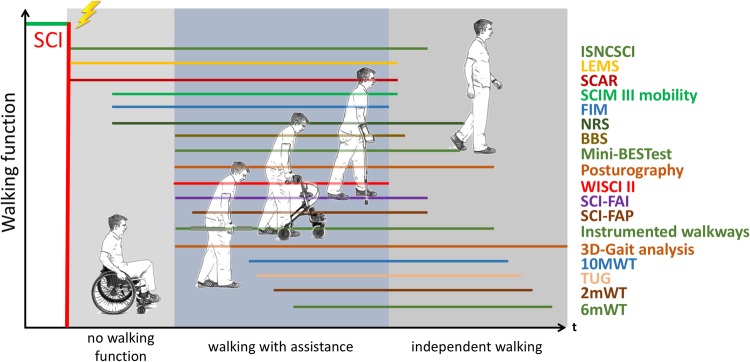
Range of walking deficit over which each “well-reviewed” LE-outcome measure can be applied. There is currently no validated outcome measure that can cover the whole spectrum of SCI from the most severely to mildly affected patients. 3D-Gait analysis consists of kinematics, kinetics and electromyography

This manuscript emphasises key recommendations of the workshop participants for selection of LE outcome measures to assess LE function. LE function was not only limited to ambulatory function (here defined as the ability to perform a given walking task, e.g., overground walking or stair climbing) as in an acute stage after SCI, walking function can often not be measured directly in the majority of patients (Fig. 1). Therefore, some of the measures discussed in this paper are applicable to individuals who are not or not yet ambulatory and are dependent on a wheelchair, while other measures are specific to any level of locomotion. Nevertheless, one should not equate walking with mobility. In fact, many people with SCI who have basic ambulatory skills, a wheelchair may be a more effective mode of mobility. Further discussion on this point is beyond the scope of the paper. Figure 1 depicts the different possible phases of LE function after SCI ranging from no walking function to independent physiological walking and illustrates the range each outcome measure can cover.

The outcome measures discussed in this review were selected if psychometric properties (reliability, validity or responsiveness) were published for the SCI population (Supplementary Tables I–IV). The factors beyond reliability, validity and responsiveness that have to be considered before choosing a specific outcome measure for a given trial are discussed in the following section. Strengths and limitations with regard to LE function are discussed based on a focused literature review and the expertise of the workshop participants.

### Key concepts

In line with the recommendations for upper extremities outcome measures [6], the following three major areas are discussed: (1) definition of important features of clinical outcome assessments, (2) comparison of strengths and limitations of established LE outcome measures and their appropriateness for human studies, and 3) discussion of remaining gaps in knowledge.

### Important features of clinical outcome assessments

Ideally, an outcome assessment should be able to identify the structural or functional mechanism underlying the observed behavioural improvement. However, a change in performance may be achieved by recovery and/or by compensatory activities. Recovery here is defined as the “restoration of the neuromuscular system to regain function using pre-injury motor behaviours, whereas behavioural compensation refers to the use of atypical motor patterns, behaviours, body segments, technology, and/or assistive devices to make up for neurologic deficits post-injury to accomplish the same or similar functional tasks” [7].

Unfortunately, most outcome measurement tools do not take into account how a functional improvement was achieved and therefore compensatory behaviours or assistive devices can mask a beneficial effect of a therapeutic intervention. For example, a patient can walk 10 meters before and after an intervention with no change in walking speed, but initially the walking was assisted with a walking aid and characterized by a pathological movement pattern; whereas, after the intervention, the walking aids were not needed and walking was performed with a more physiological movement pattern. In such a case, considering only the timed walking speed (an objective linear measure) would not accurately reveal the beneficial effect on the quality of walking. At this time, the established clinical LE outcome tools cannot accurately track all quantitative or qualitative aspects of LE motor function, nor can they reveal the underlying mechanisms leading to a change in LE motor performance. A combination of clinical LE outcome tools, often including biomechanical analyses (e.g., kinematics and kinetics) can provide more precise information about the efficiency and quality of LE movement patterns. Nonetheless, emphatic conclusions about the underlying mechanisms contributing to any LE change remain elusive [8].

Gait impairments in persons with SCI typically result from neuromuscular changes with consecutive compromised balance control, walking speed, diminished endurance and impaired gait quality caused by LE weakness, spasticity and/or sensory deficits. These limitations can be assessed by established or evolving LE outcome measures. Standard assessments include the following:

- clinical evaluations
- balance and stability measures
- time or distance measures
- gait quality assessment
- activities of daily living scales
- assessment of the dependency on walking aids
- three-dimensional (3D) gait analysis (kinetics, kinematics and EMG).

The most basic distinction among these measures is their level of measurement or scale of measure. The scale types describe the nature of information that a given value assigns to a variables [9]. These scales types range from continuous linear (interval or ratio) data to ordinal outcomes. Each scale type has specific advantages and limitations, but the main distinction is the type, accuracy and strength of the statistical analysis that can be applied based on the measurement scale. This creates challenges in interpreting statistical results across the outcome measures, e.g., many outcome measures in SCI are ordinal or rank ordered, but the intervals between rankings are unknown or unequal. Therefore, from a statistical point of view, continuous outcome measures are considered to be superior to ordinal outcome measures as they allow for parametric statistical analysis. However, the selection of outcome measure for a given trial depends also on the available time and equipment, focus of the assessment tool, and the goals of the trial phase. Table 1 summarizes some of the global strengths and limitations of LE outcome measures.

**Table 1:** Assessment of lower extremities function in SCI: global strengths and limitations of outcome measures

Scale		Strengths	Limitations
Continuous	Advanced clinical diagnostic measures (e-phys)	◦ Detailed analysis ◦ Discrimination of extent and injury subtypes (e.g., cyst, central cord syndrome,…) ◦ Can identify mechanisms underlying gait dysfunction ◦ Can produce linear measures	◦ requires expensive equipment and skilled examiner ◦ Do not assess walking function directly ◦ Complex post-processing
	Timed measures (10MWT, 6mWT, TUG)	◦ Simple and unidimensional ◦ Readily quantified ◦ Requires limited training ◦ Published norms available ◦ Often used in clinical trials	◦ Do not identify mechanisms underlying gait dysfunction ◦ Cannot discriminate compensatory strategies
	Spatiotemporal gait analysis and posturography	◦ Identify mechanisms underlying gait dysfunction ◦ Provide precise electrophysiological, kinematic, kinetic, and spatiotemporal data	◦ Requires mostly expensive equipment and skilled examiner ◦ Complex post-processing ◦ Limited to a few specialized laboratories ◦ Limited clinical impact
Ordinal	Standard clinical measure (ISNCSCI)	◦ Gold standard in clinical trials ◦ Minimal equipment (g-Tip, safety pin) required	◦ Requires a skilled examiner ◦ limited information about mechanisms underlying gait dysfunction ◦ Limited accuracy and sensitivity ◦ non-linear ordinal measures
	Gait quality measures (NRS, SCI-FAI, SCI-FAP)	Can identify mechanisms underlying gait dysfunction require limited equipment	◦ Limited precision ◦ Require skilled examiners ◦ Can be affected from floor/ ceiling effects
	Clinical LE outcome measures (WISCI II, SCIM III, FIM, BBS, Mini-BESTest)	◦ Can document the use of assistive devices ◦ Require limited time to administer ◦ Can be assessed in clinic and in community	◦ Require assessment training ◦ No or limited information about mechanisms underlying gait dysfunction ◦ Can be affected from floor/ ceiling effects

Modified after Cameron et al. [47]

*ISNCSCI* International standards for neurological classification of spinal cord injury, 10MWT ten meter walk test, *6mWT* six minute walk test, *TUG* timed up and go test, *SCAR* spinal cord ability ruler, *WISCI II* walking index for spinal cord injury II, *SCIM III* spinal cord independence measure III, *FIM* functional Independence Measure, *SCI-FAI* spinal cord injury functional ambulation inventory, *SCI-FAP* Spinal cord injury functional ambulation profile, *BBS* Berg Balance Scale, *NRS* neuromuscular recovery score

The International Classification of Functioning, Disability and Health (ICF) provides a standard language and framework for the description of health and health-related states [10]. The ICF consists of domains of function across a continuum from “body structure/function” through “activity” to “participation”. Outcome measures are expected to align with one or more of the domains along this continuum/spectrum. The ICF is therefore a useful framework to group LE assessment tools according to their potential use in different trial phases (Table 2).

**Table 2:** Framework for LE efficacy outcome measures and clinical trials outcomes

	Clinical trials	Categories of outcome measures	Influencing factors of outcomes	Outcome measures		
				Phase I	Phase II (a/b)	Phase III
Outcome measures according to ICF domains (independent of expected outcome levels)	Body structure & function	Neurological or physiological measures (or impairment)	◦ CNS integrity ◦ Neural circuits (sensory/motor)	◦ ISNCSCI ◦ e-phys/ EMG, Kinematics, Kinetics ◦ MRI	◦ ISNCSCI ◦ e-phys/ EMG, Kinematics, Kinetics ◦ MRI ◦ SCAR	◦ ISNCSCI
	Activity	Measures of capacity and performance (or limitation)	◦ CNS integrity ◦ Neural circuits ◦ Adaptive behaviours ◦ Rehabilitation ◦ Psychology	na	*Timed tests:* ◦ 10 MWT ◦ 6 minWT ◦ TUG *Non-timed tests:* ◦ SCAR ◦ WISCI II ◦ SCIM III items (mobility) ◦ SCI-FAI ◦ SCI-FAP ◦ NRS *Balance/ Trunk control:* ◦ Berg Balance Scale ◦ Mini-BESTest	*Timed tests:* ◦ 10 MWT ◦ 6 minWT ◦ TUG *Non-timed tests:* ◦ SCAR ◦ WISCI II ◦ SCIM III items (mobility) ◦ SCI-FAI ◦ SCI-FAP
	Participation	Quality of life measures (or restriction)	◦ Adaptive behaviors ◦ Rehabilitation ◦ Psychology ◦ Mobility ◦ Community/family support ◦ Finances ◦ Work/school	na	na	◦ Patient reported outcome (have not been used as primary outcome to date)

Adapted from Steeves et al. [11]. The following outcome measures provide additional insights and have been attracting attention in the field of SCI, but are yet less fully explored and require further broader application: 2 minWT; SCI-FAI; SCI-FAP; SCAR; NRS; Mini-BESTest

At the domain of body structure and function outcome, measures become related to neurological or physiological parameters, whereas for the activity domain outcome, measures are associated to functional capacity, and at the participation domain to quality of life. These domains are influenced by an increasing number of independent variables in the transition from body structure/ function through activity to participation. These independent variables will likely influence outcome assessments and change measures to an unassigned extent in a subject with SCI [11]. The greater the number of uncontrolled variables, the more difficult it is to draw a definitive conclusion from any change in an outcome variable as to what extent it may accurately reflect a beneficial effect of any experimental intervention. Although it is hoped that an experimental treatment will provide improved benefits across many domains, including participation (e.g. overall quality of life), it is the most difficult domain to assess due to the influence of a large number of independent variables that are impossible to control for or eliminate. Thus, participation and quality-of-life outcomes have not as yet been used as primary endpoints for clinical trials in SCI [12].

### Strengths and limitations of established LE outcome measures and their appropriateness for human studies

A number of established and emerging outcome measures are available to assess LE function after SCI. They must consider several domains like posture, strength, balance, adaptability of gait to environmental requirements/ factors and of course walking performance itself. They were developed for different applications, such as classification, monitoring of clinical improvements or attempting to explain the underlying mechanisms contributing to functional progress. They assess factors from impairments (e.g., strength or sensation) to capacity (e.g., gait quality) and performance (e.g., walking speed). As a consequence, the data from outcome measurement tools come in a number of different forms or scales.

### Non-ambulatory measures

A big challenge in assessing gait is that SCI therapies targeting neuro-recovery (or neuro-protection) are designed to be applied in an acute stage of SCI meaning within hours or days after injury. It is obvious that, at this early stage of SCI, walking function cannot be measured directly in the majority of patients, therefore, outcome measures that can be applied early after SCI and indirectly assess walking function, may be used to predict walking function.

The “International Standards for Neurological Classification of Spinal Cord Injury” (ISNCSCI) is considered to be the gold standard for the assessment of location, severity and extent of SCI. It consists of a motor (manual test of arm and leg key muscles) and a segmental sensory (light touch, pinprick) evaluation. On the basis of these assessments, injury severity is classified into complete (ASIA Impairment Scale A or AIS A) or incomplete SCI (AIS B/ C/ D), and the most caudal normal spinal levels are determined (motor/sensory and overall single neurological level of injury). ISNCSCI does not directly assess walking function. However, as it describes the severity of a lesion, it shows good correlation (e.g., lower extremity motor score, LEMS) with functional outcome measures such as the 10 MWT [13], 6 mWT [14] and WISCI [15]. The walking function at 1 year post injury can be likely predicted by the AIS classification acquired within 2 weeks of initial injury [16].

The “LEMS” is part of the ISNCSCI protocol [17]. Voluntary muscle force of 10 key leg muscles (5 on each side) are scored on a 6 point ordinal scale from 0 (none) to 5 (normal) with a maximum combined score of 50 points. Correlations between LEMS and LE capabilities have been validated by timed walking assessments and with the Spinal Cord Ability Ruler (SCAR) [18–20]. In addition, several studies have identified scores of individual muscles that are strongly predictive of walking ability [21]. However, LEMS shows ceiling effects in good walkers and limited responsiveness to change at scores greater than three [22–24]. Therefore, LEMS has limited ability to detect subtle changes.

“Foot control” has been shown to be a sensitive indicator to distinguish between muscle weakness and impairment of dexterity after incomplete SCI [25, 26]. It can be assessed in a supine position and the timing of ankle dorsi- to plantarflexion is tested by means of auditory-paced movements at three frequencies. Assessing voluntary foot control (differentiate between impaired muscle force and dexterity) could allow prediction of walking function but requires further research to be applicable as a clinical outcome measure.

The first motor tasks performed with patients after SCI are usually standing tasks. The ability to stand and maintain balance are requirements for walking function. Therefore, the assessment of balance could be very useful in predicting walking function [27, 28]. The “Berg Balance Scale“ (BBS) measures static and dynamic standing balance [29] and is an established measure for people with SCI [30]. Static and dynamic balance tasks (14 items) of varying difficulty are performed and items are scored on a scale from 0 to 4 points. A maximum score of 56 points can be reached, with higher values, indicating better balance performance. Although the BBS does not measure walking function directly, it is correlated with walking performance after SCI [30]. BBS shows ceiling effect in SCI subjects with AIS D who are community ambulators [30], and may be more suitable for patients with limited walking ability [31]. Recently, two new assessments have been validated for patients with SCI that should overcome the limitations the BBS has in community ambulators. The Mini-BESTest [31] and the Community Balance and Mobility Scale [32] are both outcome measures that assess balance in higher-functioning patients with SCI and show no ceiling effect in these individuals. However, in both assessments further evaluations of the psychometric properties are needed to establish them as measures in SCI.

Balance is a determinant in gait recovery and recent work has used neurophysiological approaches to assess impairment to a neuronal pathway underlying balance control, namely the vestibulospinal tract (VST). Using galvanic vestibular stimulation, correlations were drawn between impairment of the VST and balance deficits [33, 34]. As the BBS shows a clear ceiling effect [30], VST assessment could be complementary to clinical assessment of balance.

The “Posturography” is used to measure stability and balance in individuals with SCI who are able to stand and balance independently. Depending on the level of information that should be derived from this measure (e.g., biomechanical model of the subject measured vs. simple ground reaction forces), this method can require similar tools as the 3D gait analysis (see below) or just force plates. It is accurate and provides very detailed and insightful information about the subject’s ability to maintain stability during standing. However, it requires specific and expensive equipment, sophisticated computer algorithms, and highly trained examiners. It is therefore only available in specialized study centres and plays a limited (secondary or exploratory) role as an outcome tool in clinical studies.

### Assessment of locomotion

There are a lot of established outcome measures to assess locomotor ability in patients with SCI. The most basic distinction among these measures is that between continuous (i.e., quantitative), ordinal (semi-quantitative) and categorical data.

### Continuous (interval) outcome measures

Timed measures such as the 10 meter walk test (10MWT), six-minute walk test (6mWT), and timed up and go test (TUG) are the most established outcome measures in SCI using a continuous linear scale. They are used to easily assess important features of gait, including speed, endurance, turns and the adjustment from sitting/standing to walking. They only directly measure speed, but this is affected by the other variables to a greater or lesser extent depending on the duration and complexity of the test. Timed tests need minimal equipment, little time to administer, little advanced training from the assessor, and can usually be assessed in either an in-patient clinical setting or an out-patient community environment. In all of these cases, patients must be able to stand and walk with or without assistive devices; therefore, all assessments show floor effects for subjects who are unable to stand or walk. They are not appropriate if a person requires any external physical assistance to advance the legs to take a step. Normative data for the 10 MWT [30, 35], 6 mWT [36] and TUG [30] are published and enable comparison of people living with SCI and uninjured control subjects [37, 38]. The tests have been shown to be reliable, valid and responsive [13]. Reliability can be increased if patients are allowed to perform a test trial before the actual measurement [14]. However, one main drawback is that timed walking test cannot characterize aberrant motor behaviours underlying any gait dysfunction that are not directly affecting walking speed.

The “10MWT“ is used to assess gait speed. The walking distance is 14 m to accommodate 2 m for acceleration at the beginning and 2 m for deceleration at the end. Speed is calculated from the time it takes for a patient to walk the intermediate 10 m. The test can be conducted at a preferred walking speed or at the person’s fastest (but safe) speed. The assessment does not discriminate the amount of assistance provided by a walking aid, though its use should be recorded and may be kept consistent in repeated measures. It is considered that faster walking speed is a surrogate measure for an overall improvement in LE motor function and performance [39]. However, it is not clear how to define a meaningful walking speed for daily living. Different walking speeds have been recommended to discriminate between functional walking categories after SCI (indoor walkers >0.15 ± 0.08 m/s, assisted walkers >0.44 ± 0.14 m/s and independent walkers >0.70 ± 0.13 m/s) [35]. The speed needed to safely cross a street at many crosswalks was defined as 0.6 m/s [40], but crosswalk timers vary by region and sometimes require a faster walking velocity. Independent living has been correlated with a walking speed above 1.0 m/s for elderly people [41].

The “6mWT“ measures sustained walking speed and is used to assess endurance, fatigability and cardiovascular fitness [39]. The distance traversed at a preferred or fastest walking speed is measured over 6 min. The standardization of the 6 mWT is difficult and often lacking, as it depends on the length of the corridor or gymnasium a person uses to complete the walk. Walking distance and speed are likely to be influenced by the number and radius of the turns a person has to navigate during 6 min [42]. As there are many sources of variability, study instructions should be standardized for all participants. In addition, to familiarize the participant with the task, while potentially revealing any potential physical limitations, a test assessment may be considered before taking the actual measurement [14]. Unless there are accompanying notes, the 6mWT distance measured does not reveal how the outcome was achieved (e.g., patient could walk for 3 min and then stop due to exhaustion or pain). Thus, careful notation of whether the distance was achieved in a continuous walk or in shorter bursts of activity is important. Comparison of walking over a shorter time interval may clarify whether any limitation in the 6mWT distance is due to fatigue or musculoskeletal pain (see below).

The “two minute walk test” (2mWT**)** is derived from the 6mWT and assesses performance by measuring the distance a person can walk within a less demanding period of 2 min. It is not yet a well-established assessment in SCI. The original validation study for the 6 mWT, showed that 2- and 12-minute walk tests were equally valid, and the 6-minute test was endorsed because it represented an intermediate interval [43]. The 2mWT has been used in studies of persons with SCI who have more limited walking capacity being unable to walk for 6 minutes. The 2mWT has been shown to correlate with the 6mWT in people with neuromuscular diseases [44], multiple sclerosis [45], stroke [46] and SCI [30], and is therefore considered a potential alternative to the 6mWT to describe walking capacity and endurance.

The “Timed Up and Go Test” (TUG) assesses the time needed to stand up from a chair, walk 3 m, turn around, walk back to the chair and sit down. Compared to 10 MWT and 6 mWT, TUG does not just assess walking speed or endurance. TUG is a more complex task consisting of standing up–sitting down, walking, and turns with increased dependence on balance and postural control. Thus, TUG might better reflect a broad spectrum of activities of daily living (ADL) compared to more unidimensional tests that assess only gait speed and distance.

There are few continuous (interval) outcome measures for LE function besides the timed assessments. The “SCAR“ is a new and promising, but not yet clinically established measure that scores on a continuous scale. SCAR transforms ordinal, neurological and functional activity items into a continuous (interval) scale that can be used to measure all levels and severities of SCI from initial injury to at least 1 year after SCI [20]. Although it is not simply or strictly a LE functional measure, the strength of SCAR is that it focuses on a single underlying measurement domain (construct): volitional performance. This is achieved by combining selected items from two established clinical assessments (upper extremity motor score from ISNCSCI and volitional movement items from the Spinal Cord Independence Measure (SCIM)) into a new continuous score defined and validated by Rasch analysis. The strength of SCAR is that it is highly responsive (i.e., can be used to measure change in function from the initial time of injury to at least the end of the first year after SCI) [20]. Individuals with tetraplegia as well as paraplegia and complete or incomplete SCI can be simultaneously tracked. However, persons with a central cord syndrome cannot be as accurately assessed [20].

The “3D gait analysis” is considered the gold standard for the assessment of gait [47]. 3D gait analysis usually consists of kinematic, kinetic and electromyography data. There are several different setups/methods to assess 3D gait analysis. They all depend on markers (active or passive) that are placed on a person over bony (joint) landmarks according to a predefined model and recorded with cameras, while the person is actively moving. On the basis of the position of the markers in space, a 3D model of the person’s leg movements can be reconstructed. 3D gait analysis combined with force plate measurements provides detailed quantitative continuous measures of kinematics, kinetics and spatiotemporal parameters of gait. Assessments at a standardized treadmill velocity facilitate comparison between participants, however, measures acquired during treadmill walking may not translate to real-world overground walking. Training-related changes in intralimb coordination during overground walking represent a more sensitive measure of real-world improvements in neural control of limb movement [48, 49]. Due to the high precision of the assessment method, subtle gait impairments/changes can be identified and provide insight about the mechanisms underlying any gait impairment [50, 51]. Nevertheless, 3D gait analysis requires expensive equipment, sophisticated computer algorithms, and highly trained examiners. It is therefore only available in specialized study centres and plays a limited (secondary or exploratory) role as an outcome tool in clinical studies.

Recent advances in rehabilitation robotics open the way to more sensitive measures of gait control and adaptive capacity [52]. Indeed, new testing devices are now available to test foot control during gait [53, 54]. These studies show that sensorimotor processing is different during actual movement than at rest [54] and that challenging gait by applying force fields during walking might provide a sensitive tool to assess remaining adaptive capacity [53]. Combined with laboratory gait analysis (kinematics, kinetics and EMG) [55], this approach will help in guiding therapists towards the best patient-oriented rehabilitation intervention in the near future. Furthermore, recent work combining force field adaptation during human walking with electrophysiological measures has shown the potential to also assess the central reorganization associated with motor learning [56], and thereby also identify neural structures and pathways important for locomotor recovery after injury.

The “Instrumented walkways“ offer a good alternative to the costly 3D gait analysis systems. They are portable, affordable, require no advanced training to use and provide valid and reliable spatio-temporal parameters of gait (e.g., speed, step length, stance time, swing time, single support time and base of support) [47, 57, 58]. Instrumented walkways can be combined with standardized clinical assessments such as the 10 MWT or TUG and allow identification of functional systems contributing to a patient’s gait dysfunction [47].

Wearable technologies namely “inertial measurement units”, are becoming more popular in gait analysis with technological progress as they can provide spatio-temporal parameters of gait [59]. These systems are portable, low priced and require only limited training to use. However, validity and reliability have still to be proven, and therefore application of this new technology in a clinical setting is not yet feasible.

Strengths and limitations for each continuous outcome measure are summarized in Table 3.

**Table 3:** Strength and limitations of continuous outcome measures

	Targets of assessment	Strengths	Limitations
10MWT	Ambulatory capacity (walking speed)	◦ Unidimensional continuous time scale ◦ Minimal equipment needed ◦ No advanced training needed ◦ Little time to administer ◦ Can be used in clinical setting or in the community ◦ Assesses minimal walking capacity to accomplish indoor walking (walking speed, m/s)	◦ Does not discriminate the amount of physical assistance required ◦ Test can be performed at different speeds (comfortable vs. max speed) ◦ Ceiling effects ◦ Not responsive in good walkers ◦ Patient must be able to stand and walk (with or without assistive devices)
6mWT	Ambulatory and aerobic capacity	◦ Unidimensional continuous distance scale ◦ Minimal equipment needed ◦ No advanced training needed ◦ Little time to administer ◦ Can be used in clinical setting or in the community ◦ Assesses walking and aerobic capacity	◦ Does not account for assistive devices ◦ Outcome can be influenced by aerobic capacity (e.g., stamina) ◦ Cannot discriminate between patients with low capacity ◦ Does not tell us how long a patient really walked (e.g., patient could walk for 3 min and then stop walking) ◦ Rests are normally not noted ◦ Estimate of aerobic capacity is limited if LE sensorimotor impairments impede walking
2mWT	Ambulatory capacity	◦ Same as 6mWT, but not as aerobically challenging ◦ Maybe better for elderly patients	◦ Same as 6mWT but not as well established
TUG	Standing, sitting, walking, turns while walking ambulatory capacity	◦ Continuous time scale ◦ Minimal equipment needed ◦ No advanced training needed ◦ Little time to administer	◦ Does not account for assistive devices ◦ Walking speed cannot be calculated ◦ Mixture of standing, sitting, walking and turning concept of measurement ◦ Difficult to interpret
SCAR	LE and UE activities	◦ Unidimensional (voluntary motor performance) continuous scale ◦ Clear concept of measurement ◦ Repeatedly validated with random subsets from large EMSCI^a^ database ◦ Can be assessed in all patients and at all time points (independent of severity of SCI)	◦ Yet to be established in clinical trials
Kinematics	Gait quality	◦ Unidimensional continuous scale ◦ Temporal and spacial data ◦ Can identify mechanisms underlying gait dysfunction	◦ Highly specialized assessors needed ◦ Expensive equipment needed ◦ Only available in specialized study centres
Posturography	Balance capacity	◦ Unidimensional continuous scale ◦ Temporal and spacial data ◦ Can identify mechanisms underlying gait dysfunction	◦ Highly specialized assessors needed ◦ Expensive equipment needed ◦ Only available in specialized study centres
Instrumented walkways	Gait quality	◦ Unidimensional continuous scale ◦ Temporal and spacial data ◦ Affordable	◦ Provides only limited spatial data ◦ Not established in clinical trials
Inertial based units	Gait quality	◦ Unidimensional continuous scale ◦ Temporal and spacial data	◦ Clinical validity and reliability of commercial systems not yet proven

*10MWT* ten meter walk test, *6mWT* six minute walk test, *2mWT* 2 min walk test, *TUG* timed up and go test, *SCAR* spinal cord ability ruler

^a^EMSCI: European Multicenter Study about Spinal Cord Injury

### Ordinal outcome measures

Most of the established clinical outcome measures yield ordinal data collected across multiple domains. Some of these ordinal tools have a long history for clinically describing a person’s impairment after SCI or a person’s overall functional capacity. Most of these assessment tools were not created as trial outcome measures (rather clinical descriptors), but have been repurposed as outcome measures with varying success.

The “Spinal Cord Independence Measure III” (SCIM III) is a SCI-specific disability assessment that describes the ability of a person with SCI to perform various activities of daily living (ADLs) [60]. The SCIM III subitem “mobility indoors and outdoors” is a sub-score of the larger multidimensional measure and consists of six items: mobility indoors, mobility for moderate distances (10–100 m), mobility outdoors (>100 m), stair management, transfers: wheelchair-car and transfers: ground-wheelchair. The items are scored on varying 2–9 level categorical scales with higher scores reflecting a higher level of independence. The score allows assessment of subjects with SCI across a broad range of clinical presentations (from wheelchair use to walking without aids). The SCIM III mobility sub-score shows floor effects in severely affected patients [61] and ceiling effects in good walkers, and it does not differentiate among subjects on the basis of their gait or walked distance (>100 m).

The “Functional Independence Measure” (FIM) assesses basic activities of daily living and consists of two subscales: a motor (13 tasks) and a cognitive (5 tasks) subscale [62]. All tasks are rated on a 7-point ordinal scale that ranges from total dependence to complete independence. The FIM scores range from 18 to 126 points. Two tasks are related to walking function (FIM_L_): locomotion (ambulatory or wheelchair level) and stair climbing. The FIM_L_ does not consider the use of assistive devices or braces to enable independence [63]. FIM_L_ shows ceiling effects in good walkers [63]. FIM is not a SCI-specific outcome measure, resulting in limitation in sensitivity to assess subtle (but important) changes in locomotor function.

FIM and SCIM III are multidimensional scores across a range of domains. In both assessments, only sub-items are related to locomotion (2 sub-items in FIM and 6 sub-items in SCIM III). In addition, the sub-items are not pure ambulation assessments as one option of scoring could be wheelchair use. However, it is suggested that SCIM III is more applicable in patients with SCI to assess LE function than FIM [63, 64].

The “Walking Index for Spinal Cord Injury II” (WISCI II) assesses the physical assistance (i.e., number of people) and assistive devices (i.e., walking aids) a patient needs to ambulate 10 metres [65]. The scale assesses walking function on a hierarchical scale from 0 to 20, where higher numbers indicate less impairment. The rank on the index is dependent on the amount of assistance (device, braces and physical assistance) used to ambulate 10 m on a level surface. WISCI II has clinical impact as it is widely used in the SCI population [66], however, it has some limitations. The score shows ceiling effects in patients who do not require assistive devices/physical assistance and is therefore not suitable for patients with good walking function [30, 67]. Scoring for individuals with very poor walking capacity will also generate a floor effect [68]. Like many ordinal scales, WISCI is not a linear scale because the distance between each successive ranked score might not be equal in terms of difficulty to perform. Finally, therapists’ decisions regarding which walking aids to prescribe are dependent on factors that indirectly influence walking such as balance and safety, and therefore, the assessor will affect the WISCI score by requiring walking aids for safety reasons. This is also reflected by the finding that in people with chronic SCI who are capable of ambulating at multiple WISCI levels show more efficient ambulation (e.g., higher walking speed) at self-selected WISCI [67].

The “Spinal Cord Injury Functional Ambulation Inventory” (SCI-FAI) consists of three key domains of walking function: an ordinal score that assesses the quality of gait (observational gait assessment), an ordinal score that assesses the use of assistive devices and a score that assesses temporal/distance aspects of walking (distance walked within 2 min and an ambulation classification score) [69]. Higher scores indicate higher levels of function in each subscale. Each key domain is interpreted separately as an overall composite score would result in a multidimensional score, describing different aspects of gait. SCI-FAI can only be applied in patients with SCI who can ambulate independently with or without the use of assistive devices. It shows ceiling effects in good walkers in the observational gait assessment, the assistive device score and the ambulation classification score [30]. SCI-FAI is not yet a routine clinical study outcome measure. However, it has the potential to broadly categorize the mechanisms (compensation vs. recovery) underlying improvements in LE function over time and therefore could shortly become more commonly used.

The “Spinal Cord Injury Functional Ambulation Profile” (SCI-FAP) consists of 7 timed walking tasks (Carpet, Up & Go, Obstacles, Stairs, Carry, Step, and Door) performed at comfortable walking speed [70]. Time needed to complete each task is recorded and a task score is calculated by multiplying the time by a factor quantifying the assistance needed and then normalized to the mean scores from able-bodied individuals [task score = (time × factor) / mean able-bodied time]. The maximum score is 2100 relates to less LE ability. Patients who cannot walk would score always 2100. Lower scores indicate greater LE ability (less time and assistance needed). The test does not differentiate between different levels of manual assistance or account for any bracing or orthosis used by the patients. As all sub-tasks showed high reliability and each task can be used in an independent fashion. SCI-FAP shows ceiling effects in good walkers and cannot discriminate between individuals who walk at normal speeds without devices or physical assistance [70].

The “Neuromuscular recovery scale” (NRS) classifies 14 functional performance tasks related to mobility, standing and walking [71]. It is a new outcome tool and all items are scored by comparing current performance with normative performance (assumed to be pre-injury performance), thereby describing the amount of recovery reflected in capabilities without compensatory movement assistance. A total score of 161 points can be achieved with higher values indicating higher degree of recovery. No floor or ceiling effect have been observed so far in chronic SCI of all severities [71]. NRS can detect compensatory strategies and therefore broadly categorize the mechanism underlying improvements in LE function.

Strengths and limitations for each ordinal outcome measure are summarized in Table 4.

**Table 4:** Strength and limitations of ordinal LE outcome measures

	Targets of assessment	Strengths	Limitations
ISNCSCI	Sensory and muscle strength	◦ Gold standard assessment in SCI ◦ Minimal equipment needed ◦ Can be assessed in most patients and at all time points (independent of severity of SCI)	◦ Multidimensional ordinal scale ◦ Assessor training is mandatory ◦ No assessment of walking function ◦ Can be time consuming
LEMS	Muscle strength	◦ Can be assessed in most patients and at all time points (independent of severity of SCI) unidimensional measure	◦ Ordinal scale ◦ LEMS does not always correlate with walking quality
Berg balance score	Balance trunk control	◦ Minimal equipment needed ◦ No advanced training needed	◦ Patient can maintain balance but may not walk ◦ Ordinal score ◦ Ceiling effects
Mini-BESTest	Balance trunk control	◦ Minimal equipment needed ◦ No advanced training needed	◦ Ordinal score
SCIM III (mobility items only)	Ambulatory capacity ability to climb stairs functional mobility	◦ Moderate training required ◦ Low costs ◦ Can be assessed in interview ◦ Clinically relevant and commonly used ◦ Only scale to consider real world performance	◦ Assesses walking distance in 3 broad categories (<10 m, 10–100 m, >100 m) and dependence on any assistive device ◦ Does not assess gait ◦ Ordinal score ◦ Ceiling effects
FIM (mobility items only)	Ambulatory capacity ability to climb stairs	◦ Moderate training required ◦ Minimal equipment needed	◦ Does not assess gait ◦ Not SCI specific outcome measure ◦ Low sensitivity to subtle changes ◦ Not free available
WISCI II	Dependence/independence for walking with or without assistance	◦ Low costs ◦ Complements other functional tests in LE (e.g., 10MWT, 6mWT)	◦ Ceiling effect in majority of patients (not suitable for patients with good walking function) ◦ Ordinal scale
SCI-FAI	Gait (quality of walking) assistive devices ambulatory capacity	◦ Low costs ◦ Can be assessed in clinic and in community	◦ Ceiling effects in good walkers ◦ Multidimensional ordinal scale
SCI-FAP	Ambulatory capacity	◦ Assesses walking tasks of greater complexity (e.g., different floor surfaces) ◦ Moderate training needed	◦ Multidimensional ordinal scale ◦ Specific equipment needed ◦ Ceiling effects
Neuromuscular recovery scale	Pre injury movement pattern	◦ Can differentiate between compensation and recovery	◦ Advanced training needed ◦ Body weight support treadmill needed ◦ Yet to be established

*ISNCSCI* International standards for neurological classification of spinal cord injury, *LEMS* lower extremity motor score, *WISCI II* walking index for spinal cord injury, *SCIM III* spinal cord independence measure, *SCI-FAI* spinal cord injury functional ambulation inventory, *SCI-FAP* spinal cord injury functional ambulation profile

### Advanced clinical diagnosis tools

Most clinical assessments of lower limb function may have limitations in respect of detecting subtle changes in sensorimotor function. “Clinical neurophysiological techniques” (e-phys) may provide this information as they produce quantitative measures of spinal cord function [72]. In addition, these measures can be applied in a very early stage after injury. The main purpose of e-phys measurements is to assess the extent and level of SCI. However, such measures may also prove to be predictive of functional recovery in lower and upper extremities after SCI [73, 74]. Although e-phys is only an indirect measure of lower limb function it can specify and quantify the underlying pathophysiology of gait impairments following SCI [72]. E-phys assessments require expensive equipment and highly trained examiners, and are specifically superior to predict functional outcomes compared to clinical measures where patients are not able to cooperate with the clinical assessment (i.e. unconscious or intoxicated patients) [21]. Further details about e-phys can be found elsewhere [72–74].

## Conclusions and recommendations

There is no single outcome measure that can be universally applied to all people living with SCI to track changes in LE performance regardless of severity and level of injury (Fig. 1). Walking is important to most people after SCI [75, 76] and certainly LE function shows some improvement following incomplete cervical or thoracolumbar SCI [77]. Thus, carefully tracking change in LE activities is fundamental in assessing any clinical treatment effect.

It is generally agreed that a direct measurement of the performance for an appropriate functional activity supersedes any surrogate biomarker or neurological indicator (e.g., ISNCSCI). However, for aspects of feasibility, we are limited in measuring complex LE functional activities at the acute stage after SCI. Furthermore, how to relate measures of a patient with limited LE (non-ambulatory) movement at an early stage after SCI to any eventual recovery of ambulatory ability? Thus, we often have no baseline measurement of the primary or secondary outcome measure, we would like to follow across the whole range from very acute to end of trial participation. Shakespeare’s Hamlet stated it succinctly “Ay, there’s the rub!”

The unidimensional interval SCAR was created from elements of ISNCSCI and SCIM III to overcome this obstacle; should it be independently validated in prospective clinical studies, it might become a valid and useful clinical outcome tool in SCI studies. Clinical trials in SCI would be best served by having unidimensional interval measures that can be applied at early and late time points after spinal injury for all people, regardless of level or severity of SCI.

It is reasonable to suggest that the timed walk or distance walked outcome measures (10MWT, 6mWT, TUG) are valid study metrics for people already possessing good ambulatory abilities at the beginning of a study involving individuals with chronic SCI. For example, the efficacy of a rehabilitation training study designed to improve a person’s fitness and/or walking capacity after incomplete SCI could be supported by overall improvements in walking speed or distance [35]. But these measures do not track the quality of the gait [47]. If this is important for assessing the benefits of an intervention, then the assessment of changes in items within SCI-FAI, NRS or 3D kinematic gait analysis would be useful tools.

Table 2 highlights clinical trial phases and summarizes the LE outcome measures that could be chosen for a specific trial phase. It depicts that an outcome measure has to be selected according to the therapeutic target of an intervention (level where a therapeutic effect should be achieved, e.g., body structure & function—activity—participation) and the clinical trial phase. In addition, the expected effect size of the intervention influences the selection of the outcome measures too. If high ambulatory performance is expected (e.g., in AIS D patients) outcome measures that show ceiling effects have to be avoided and vice versa if low to medium ambulatory performance is expected (e.g., in AIS A/B patients) measures showing floor effect should be avoided. ISNCSCI is the clinical gold standard and therefore recommended to be assessed in all clinical trials independent of the phase or the therapeutic target of the intervention. However, ISNCSCI does not measure ambulation directly, and therefore can only act as a surrogate measure.

What remains unresolved is the priority for people living with SCI related to walking function. Do they want faster walking speed with greater distances travelled or do they wish for improved efficiency in their limb movements to approximate those of able-bodied people? Undoubtedly, most people living with SCI want both, but which elements are more imperative or more likely to be affected by interventions being tested?

In summary, there are a number of sensitive, accurate, and responsive outcome tools measuring both quantitative and qualitative aspects of LE function. The field is well served. The remaining obstacle is refinement of the tools, so they can be used across all time points after SCI, regardless of the level or severity of spinal injury.

## Disclaimer

The views expressed here are those of the authors, which do not necessarily represent those of their employers.

## Electronic supplementary material

Table I: Reliability and validity of continuous outcome measures

Table II: Reliability and validity of ordinal outcome measures

Table III: Responsiveness and minimal detectable change (MDC) of continuous outcome measures

Table IV: Responsiveness and minimal detectable change (MDC) of ordinal outcome measures

References (for supplementary tables)
