# Supplementary material for: Lower extremity outcome measures: considerations for clinical trials in spinal cord injury
Source: Spinal Cord. 2018 Apr 27;56(7):628–42. doi: 10.1038/s41393-018-0097-8 (PMC6131138; doi:10.1038/s41393-018-0097-8)
Supplement: Supplementary file 1 — Table I: Reliability and validity of continuous outcome measures [file 41393_2018_97_MOESM1_ESM.pdf]

Supplementary table I: Reliability and validity of continuous outcome measures

| | Scoring dimension | Reliability | | | Validity |
|---|---|---|---|---|---|
| | | Test-retest | Intra-rater | Inter-rater | |
| 10MWT | Gait speed (m/s) | | r=0.983 (n=22) [1]<br>ICC=0.98-0.99 (n=37) [2] | r=0.974 (n=20) [1]<br>ICC=0.95-0.98 (n=37) [2] | TUG: r=0.89 (n=70) [1]<br>6MWT: ρ=-0.95 (n=62) [1]<br>WISCI II: ρ=-0.68 (n=67) [1] |
| 6mWT | Distance (m) | | r=0.981 (n=22) [1]<br>ICC=0.99 (n=37) [2] | r=0.970 (n=20) [1]<br>ICC=0.99 (n=37) [2] | 10MWT: ρ=-0.95 (n=62) [1]<br>TUG: ρ=-0.88 (n=62) [1]<br>WISCI II: ρ=0.60 (n=60) [1] |
| 2mWT | Distance (m) | | r=0.97 (n=46) [3][a] | | 10mWT: r=0.932 (n=32) [4]<br>TUG: r=-0.623 (n=32) [4]<br>WISCI II: ρ=0.749 (n=32) [4] |
| TUG | Time (s) | | r=0.979 (n=22) [1] | r=0.973 (n=20) [1] | 10MWT: r=0.89 (n=70) [1]<br>6MWT: ρ=-0.88 (n=62) [1]<br>WISCI II: ρ=-0.76 (n=67) [1] |
| SCAR | Arbitrary unit<br>linear unit | | | | Internally validated with second independent data set.<br>Pearson separation index = 0.97 [5] |
| 3D gait analysis | Spatiotemporal | Sagittal plane: ICC=0.83-0.99 (n=15) [6]<br>Frontal plane: ICC=0.90-0.97 (n=15) [6]<br>Transverse plane: ICC=0.50-0.98 (n=15) [6] | | | Considered to be the gold standard for determining lower limbs joint kinematics during walking [7] |
| Instrumented walkways | Spatiotemporal<br>(s, m, m/s) | ICC=0.83-0.97 (n=16) [8] | | | CSA®-GAITRait®: 0.44-0.99 (n=25) [9][b] |
| Inertial measurement units | Spatiotemporal | ICC=0.55-0.94 (n=10) [10][b] | | | 3D gait analysis: r=0.93-1.0 (n=1166 steps) [10][c] |

[a] no values for patients with SCI available. Values from subjects with neurological movement disorders.
[b] no values for patients with SCI available. Values from control subjects.
[c] no values for patients with SCI available. Values from mixed group (control (n=11) and PD (n=4) subjects).
ρ: Spearman's rank correlation coefficient
r: Pearson correlation coefficient
ICC: intraclass correlation coefficient
*10MWT* ten meter walk test, *6mWT* six minute walk test, *2mWT* two minute walk test, *TUG* timed up and go test, *SCAR* spinal cord ability ruler
