# Supplementary material for: Lower extremity outcome measures: considerations for clinical trials in spinal cord injury
Source: Spinal Cord. 2018 Apr 27;56(7):628–42. doi: 10.1038/s41393-018-0097-8 (PMC6131138; doi:10.1038/s41393-018-0097-8)
Supplement: Supplementary file 2 — Table II: Reliability and validity of ordinal outcome measures [file 41393_2018_97_MOESM2_ESM.pdf]

Supplementary table II: Reliability and validity of ordinal outcome measures

	**Scoring dimension**	**Reliability**			**Validity**
		Test-retest	Intra-rater	Inter-rater	
ISNCSCI	Neurological measures		Upper extremity motor score: ICC=0.98 (n=2; 5 raters) [11]<br>Light touch: ICC=0.99 (n=4; 12 raters) [11]<br>Pin prick: ICC=0.99 (n=4; 12 raters) [11]	Total motor score: ICC=0.99 (n=43) [12]<br>Light touch: ICC=0.997 (n=30) [12]<br>Pin prick: ICC=0.988 (n=30) [12]<br>Total motor score: ICC=0.98 (n=16) [11]<br>Upper extremity motor score: ICC=0.96 (n=16) [11]<br>Lower extremity motor score: ICC=0.98 (n=6) [11]<br>Light touch: ICC=0.96 (n=16) [11]<br>Pin prick: ICC=0.89 (n=16) [11]	6MWT:<br>Total motor score: r=0.64 (n=152) [13]<br>LEMS: r=0.70 (n=152) [13]<br>UEMS: r=0.24 (n=152) [13]<br><br>10mWT:<br>Total motor score: r=0.63 (n=152) [13]<br>LEMS: r=0.69 (n=152) [13]<br>UEMS: r=0.24 (n=152) [13]<br><br>BBS:<br>Total motor score: r=0.75 (n=152) [13]<br>LEMS: r=0.79 (n=152) [13]<br>UEMS: r=0.30 (n=152) [13]
SCIM III (mobility indoors/outdoors)	ambulatory capacity			ICC=0.967 (n=372) [14]	Rasch infit=0.94 (n=425) [15]<br>Rasch outfit=0.89 (n=425) [15]
FIM (locomotor items)	ambulatory capacity			Mobility: r=0.733 (n=40) [16]<br>Locomotion: r=0.454 (n=40) [16]<br>Walk/wheelchair item: r=0.62 (n=57) [17]<br>Stairs item: r=0.32 (n=57) [17]	Walk/wheelchair<br>Rasch infit=1.29 (n=358) [18]<br>Rasch outfit=1.53 (n=358) [18]<br>Stairs<br>Rasch infit=3.56 (n=358) [18]<br>Rasch outfit=4.70 (n=358) [18]

	Scoring dimension	Reliability			Validity
		Test-retest	Intra-rater	Inter-rater	
WISCI II	dependence/<br>independence for walking	Self-selected WISCI:<br>ICC=0.994 (n=63) [19]<br>maximum WISCI:<br>ICC=0.995 (n=63) [19]	Self-selected WISCI:<br>ICC=1.00 (n=26) [20]<br>maximum WISCI:<br>ICC=1.00. (n=26) [20]	Self-selected WISCI:<br>ICC=1.00 (n=26) [20]<br>maximum WISCI:<br>ICC=0.98 (n=26) [20]	Self-selected WISCI<br>MMT: ρ=0.647 (n=75) [19]<br>Speed: ρ=0.584 (n=75) [19]<br><br>maximum WISCI<br>MMT: ρ=0.663 (n=75) [19]<br>Speed: ρ=0.693 (n=75) [19]
SCI-FAI	quality of walking<br>assistive devices<br>ambulatory capacity		ICC=0.850-0.956 (n=22) [21]	ICC=0.703–0.840 (n=22) [21]	Gait speed: r=-0.70 - -0.74 (n=22) [21]<br>Self-report walking mobility: r=0.697 (n=22) [21]
SCI-FAP	ambulatory capacity	Total score ICC = 0.983 (n=32) [22]<br>Individual items<br>ICC=0.952-0.998 (n=32) [22]		Total score: ICC= 1.000 (n=32) [22]<br>Individual items:<br>ICC=0.994-1.000 (n=32) [22]	Total score (n=32) [22]<br>10mWT: r=-0.59<br>6mWT: r=-0.59<br>Self-selected WISCI II: r=-0.68<br>max WISCI II: r=-0.70
Berg Balance Scale	balance<br>trunk control			ICC = 0.953 (n=40) [23]	2mWT: ρ=0.781 (n=32) [4]<br>10mWT: ρ=0.792 (n=32) [4]<br>TUG: ρ=-0.815 (n=32) [4]<br>WISCI II: ρ=0.816 (n=32) [4]
Neuromuscular recovery scale	pre injury movement pattern	Total score:<br>ρ=0.99 (n=69) [24]<br>Individual items:<br>ρ=0.84-0.99 (n=69) [24]		Total score: ICC=0.97 (n=10) [25]<br>Individual items:<br>ICC=0.82-0.98 (n=10) [25]	Individual items:<br>Rasch infit=0.47-1.50 (n=188) [26]<br>Rasch outfit=0.47-1.40 (n=188) [26]

ρ: Spearman’s rank correlation coefficient
r: Pearson correlation coefficient
ICC: intraclass correlation coefficient
ISNCSCI International standards for neurological classification of spinal cord injury, *SCIM III* spinal cord independence measure III, *FIM* functional independence measure, *WISCI II* walking index for spinal cord injury, *SCI-FAI* spinal cord injury functional ambulation inventory, *SCI-FAP* spinal cord injury functional ambulation profile
