# Supplementary material for: Lower extremity outcome measures: considerations for clinical trials in spinal cord injury
Source: Spinal Cord. 2018 Apr 27;56(7):628–42. doi: 10.1038/s41393-018-0097-8 (PMC6131138; doi:10.1038/s41393-018-0097-8)
Supplement: Supplementary file 3 — Table III: Responsiveness and minimal detectable change (MDC) of continuous outcome measures [file 41393_2018_97_MOESM3_ESM.pdf]

Supplementary table III: Responsiveness and minimal detectable change (MDC) of continuous outcome measures

Outcome measure	Responsiveness	MDC
10mWT	Mean change (m/s) between 1 and 3 months postinjury (P<0.001): Effect size: 0.90 [a] (n=18) [27]<br>Mean change (m/s) between 3 and 6 months postinjury (P<0.001): Effect size: 0.46 [a] (n=18) [27]<br><br>Assessed before and after at least 20 sessions of locomotor training<br>AIS A-D (n=152, median time post SCI: 0.9 years) SRM: 0.51 [13]<br>AIS C (n=39) SRM: 0.50 [13]<br>AIS D (n=49) SRM: 0.98 [13]	0.13 m/s [15]
6mWT	Mean change between 1 and 3 months postinjury (P<0.001): Effect size: 0.91 [a] (n=18) [27]<br>Mean change between 3 and 6 months postinjury (P<0.001): Effect size: 0.49 [a] (n=18) [27]<br><br>Assessed before and after at least 20 sessions of locomotor training<br>AIS A-D (n=152, median time post SCI: 0.9 years) SRM: 0.48 [13]<br>AIS C (n=39) SRM: 0.50 [13]<br>AIS D (n=49) SRM: 0.83 [13]	45.8 m [15]
2mWT	NA	NA
TUG	NA	10.8s [15]
SCAR	NA	NA
3D gait analysis	NA	NA
Instrumented walkways	NA	Speed: 0.17 m/s (n=16) [8]<br>Step length: 0.11-0.17 m (n=16) [8]<br>Cadence: 13 steps/min (n=16) [8]<br>Single limb support: 0.22-0.28 s (n=16) [8]<br>Double limb support: 0.53-0.69 s (n=16) [8]
Inertial measurement units	NA	NA

[a] Effect size = Hedge’s g (unbiased) calculated from published data
MDC: minimal detectable change
NA: not available in SCI
SRM: standardized response mean
10MWT ten meter walk test, *6mWT* six minute walk test, *2mWT* two minute walk test, *TUG* timed up and go test, *SCAR* spinal cord ability ruler
