# Supplementary material for: Lower extremity outcome measures: considerations for clinical trials in spinal cord injury
Source: Spinal Cord. 2018 Apr 27;56(7):628–42. doi: 10.1038/s41393-018-0097-8 (PMC6131138; doi:10.1038/s41393-018-0097-8)
Supplement: Supplementary file 4 — Table IV: Responsiveness and minimal detectable change (MDC) of ordinal outcome measures [file 41393_2018_97_MOESM4_ESM.pdf]

Supplementary table IV: Responsiveness and minimal detectable change (MDC) of ordinal outcome measures

Outcome measure	Responsiveness	MDC
LEMS (ISNCSCI)	Assessed before and after at least 20 sessions of locomotor training<br>AIS A-D (n=152, median time post SCI: 0.9 years) SRM: 0.23 [13]<br>AIS C (n=39) SRM: 0.72 [13]<br>AIS D (n=49) SRM: 0.16 [13]	AIS total motor score: 1.87 [28]
SCIM III (mobility indoors/outdoors)	Responsiveness compared with FIM (McNemar test): $SCIM_{mob}$ III identified more changes than FIM_{mob} (P<0.01)[14,29]	$SCIM_{mob}$ III: 1.96 [30]
FIM (locomotor items)	Discrimination between paraplegia and tetraplegia (P<0.001): Effect size: 0.38 [15]<br>Score between admission and discharge from neurorehabilitation: FIM(walk/wheelchair): Effect size: 0.82 [a][31]<br>FIM (stair): Effect size: 0.76 [a][31]	FIM_L: 4.4 [15]
WISCI II	Discrimination between 1 and 3 months postinjury (P<0.001) Effect size: 2.05 [15]<br>Discrimination between 3 and 6 months postinjury (P=0.004) Effect size: 0.73 [15]	Self-selected WISCI II: 0.785 (n=63) [19]<br>Maximum WISCI II: 0.597 (n=63) [19]
SCI-FAI (Gait score)	Mean change after an experimental gait intervention: 44.7% (P<0.001) (n=22) [21]	Gait score: 1.9 [15]
SCI-FAP	After two months of training (precision or endurance) SCI-FAP is responsive to change only in patients who walk at slow speeds (<0.5 m/s) [32]	SCI-FAP score: 95.7 [32]<br>SCI-FAP time (s): 114.2 [32]
Berg Balance Scale	Assessed before and after at least 20 sessions of locomotor training<br>AIS A-D (n=152, median time post SCI: 0.9 years) SRM: 0.59 [13]<br>AIS C (n=39) SRM: 0.65 [13]<br>AIS D (n=49) SRM: 0.91 [13]	NA
NRS_{LE}	Assessed before and after at least 20 sessions of locomotor training (n=72) $NRS_{LE:}$ ARM=1.07 [33]	NRS_{LE}: 3 [33]

[a] data from a heterogenic group of patients with Spinal Cord Injury, Multiple Sclerosis and Stroke
ARM: adjusted response mean
MDC: minimal detectable change
NA: not available in SCI
SRM: standardized response mean
LEMS lower extremity motor score, *ISNCSCI* International standards for neurological classification of spinal cord injury, *SCIM III* spinal cord independence measure III, *FIM* functional independence measure, *WISCI II* walking index for spinal cord injury, *SCI-FAI* spinal cord injury functional ambulation inventory, *SCI-FAP* spinal cord injury functional ambulation profile, NRS_{LE} Neuromuscular recovery scale trunk and lower-extremity items
