# Supplementary material for: Lower extremity outcome measures: considerations for clinical trials in spinal cord injury
Source: Spinal Cord. 2018 Apr 27;56(7):628–42. doi: 10.1038/s41393-018-0097-8 (PMC6131138; doi:10.1038/s41393-018-0097-8)
Supplement: Supplementary file 5 — References (for supplementary tables) [file 41393_2018_97_MOESM5_ESM.pdf]

References for supplementary tables I-IV

1 Van Hedel HJ, Wirz M, Dietz V. Assessing walking ability in subjects with spinal cord injury: Validity and reliability of 3 walking tests. *Arch Phys Med Rehabil* 2005; **86**: 190–196.

2 Scivoletto G, Tamburella F, Laurenza L, Foti C, Ditunno JF, Molinari M. Validity and reliability of the 10-m walk test and the 6-min walk test in spinal cord injury patients. *Spinal Cord* 2011; **49**: 736–740.

3 Rossier P, Wade DT. Validity and reliability comparison of 4 mobility measures in patients presenting with neurologic impairment. *Arch Phys Med Rehabil* 2001; **82**: 9–13.

4 Lemay J-FF, Nadeau S. Standing balance assessment in ASIA D paraplegic and tetraplegic participants: Concurrent validity of the Berg Balance Scale. *Spinal Cord* 2010; **48**: 245–250.

5 Reed R, Mehra M, Kirshblum S, Maier D, Lammertse D, Blight A *et al.* Spinal cord ability ruler: an interval scale to measure volitional performance after spinal cord injury. *Spinal Cord* 2017; **55**: 730–738.

6 Wedege P, Steffen K, Strøm V, Opheim AI. Reliability of three-dimensional kinematic gait data in adults with spinal cord injury. *J Rehabil Assist Technol Eng* 2017; **4**: 205566831772999.

7 Cameron MH, Wagner JM. Gait abnormalities in multiple sclerosis: Pathogenesis, evaluation, and advances in treatment. *Curr Neurol Neurosci Rep* 2011; **11**: 507–515.

8 Nair PM, Hornby T G, Behrman AL. Minimal detectable change for spatial and temporal measurements of gait after incomplete spinal cord injury. *Top Spinal Cord Inj Rehabil* 2012; **18**: 273–81.

9 Bilney B, Morris M, Webster K. Concurrent related validity of the GAITRite walkway system for quantification of the spatial and temporal parameters of gait. *Gait Posture* 2003; **17**: 68–74.

10 Kluge F, Gaßner H, Hannink J, Pasluosta C, Klucken J, Eskofier BM. Towards Mobile Gait Analysis: Concurrent Validity and Test-Retest Reliability of an Inertial Measurement System for the Assessment of Spatio-Temporal Gait Parameters. *Sensors (Basel)* 2017; **17**. doi:10.3390/s17071522.

11 Marino RJ, Jones L, Kirshblum S, Tal J, Dasgupta A. Reliability and repeatability of the motor and sensory examination of the international standards for neurological classification of spinal cord injury. *J Spinal Cord Med* 2008; **31**: 166–170.

12 Savic G, Bergström EMK, Frankel HL, Jamous MA, Jones PW. Inter-rater reliability of motor and sensory examinations performed according to American Spinal Injury Association standards. *Spinal Cord* 2007; **45**: 444–451.

13 Harkema S, Shogren C, Ardolino E, Lorenz D. Assessment of functional improvement without compensation for human spinal cord injury: extending the Neuromuscular Recovery Scale to the upper extremities. *J Neurotrauma* 2016; **2190**: 1–58.

14 Itzkovich M, Gelernter I, Biering-Sorensen F, Weeks C, Laramee MT, Craven BC *et al.* The Spinal Cord Independence Measure (SCIM) version III: Reliability and validity in a multi-center international study. *Disabil Rehabil* 2007; **29**: 1926–1933.

15 Lam T, Noonan VK, Eng JJ, SCIRE Research Team. A systematic review of functional ambulation outcome measures in spinal cord injury. *Spinal Cord* 2008; **46**: 246–54.

16 Grey N, Kennedy P. The functional independence measure: A comparative study of clinician and self ratings. *Paraplegia* 1993; **31**: 457–461.

17 Segal ME, Ditunno JF, Staas WE. Interinstitutional agreement of individual functional independence measure (Fim) items measured at two sites on one sample of sci patients. *Paraplegia* 1993; **31**: 622–631.

18 Nilsson ÅL, Sunnerhagen KS, Grimby G. Scoring alternatives for FIM in neurological disorders applying Rasch analysis. *Acta Neurol Scand* 2005; **111**: 264–273.

19 Burns AS, Delparte JJ, Patrick M, Marino RJ, Ditunno JF. The reproducibility and convergent validity of the walking index for spinal cord injury (WISCI) in chronic spinal cord injury. *Neurorehabil Neural Repair* 2011; **25**: 149–157.

20 Marino RJ, Scivoletto G, Patrick M, Tamburella F, Read MS, Burns AS *et al.* Walking index for spinal cord injury version 2 (WISCI-II) with repeatability of the 10-m walk time: Inter- and intrarater reliabilities. *Am J Phys Med Rehabil* 2010; **89**: 7–15.

21 Field-Fote EC, Fluet GG, Schafer SD, Schneider EM, Smith R, Downey PA *et al.* The spinal cord injury functional ambulation inventory (SCI-FAI). *J Rehabil Med* 2001; **33**: 177–181.

22 Musselman K, Brunton K, Lam T, Yang J. Spinal Cord Injury Functional Ambulation Profile: A New Measure of Walking Ability. *Neurorehabil Neural Repair* 2011; **25**: 285–293.

23 Wirz M, Müller R, Bastiaenen C. Falls in Persons With Spinal Cord Injury: Validity and Reliability of the Berg Balance Scale. *Neurorehabil Neural Repair* 2010; **24**: 70–77.

24 Behrman AL, Velozo C, Suter S, Lorenz D, Basso DM. Test-retest reliability of the Neuromuscular Recovery Scale. *Arch Phys Med Rehabil* 2015; **96**: 1375–84.

25 Basso DM, Velozo C, Lorenz D, Suter S, Behrman AL. Interrater Reliability of the Neuromuscular Recovery Scale for Spinal Cord Injury. *Arch Phys Med Rehabil* 2015; **96**: 1397–1403.

26 Velozo C, Moorhouse M, Ardolino E, Lorenz D, Suter S, Basso DM *et al.* Validity of the Neuromuscular Recovery Scale: A Measurement Model Approach. *Arch Phys Med Rehabil* 2015; **96**: 1385–1396.

27 van Hedel HJA, Dietz V, Curt A. Assessment of Walking Speed and Distance in Subjects With an Incomplete Spinal Cord Injury. *Neurorehabil Neural Repair* 2007; **21**: 295–301.

28 Scivoletto G, Tamburella F, Laurenza L, Molinari M. Distribution-based estimates of clinically significant changes in the International Standards for Neurological Classification of Spinal Cord Injury motor and sensory scores. *Eur J Phys Rehabil Med* 2013; **49**: 373–84.

29 Bluvshtein V, Front L, Itzkovich M, Aidinoff E, Gelernter I, Hart J *et al.* SCIM III is reliable and valid in a separate analysis for traumatic spinal cord lesions. *Spinal Cord* 2011; **49**: 292–296.

30 Scivoletto G, Tamburella F, Laurenza L, Molinari M. The spinal cord independence measure: how much change is clinically significant for spinal cord injury subjects. *Disabil Rehabil an Int Multidiscip J* 2013; **35**: 1808–1813.

31 Cano SJ, O'Connor RJ, Thompson AJ, Hobart JC. Exploring disability rating scale responsiveness II: Do more response options help? *Neurology* 2006; **67**: 2056–2059.

32 Musselman KE, Yang JF. Spinal Cord Injury Functional Ambulation Profile: a preliminary look at responsiveness. *Phys Ther* 2014; **94**: 240–50.

33 Tester NJ, Lorenz DJ, Suter SP, Buehner JJ, Falanga D, Watson E *et al.* Responsiveness of the Neuromuscular Recovery Scale During Outpatient Activity-Dependent Rehabilitation for Spinal Cord Injury. *Neurorehabil Neural Repair* 2016; **30**: 528–38.
